# FADD regulates adipose inflammation, adipogenesis, and adipocyte survival

**DOI:** 10.1038/s41420-024-02089-x

**Published:** 2024-07-15

**Authors:** Jianlei Tang, Yue Ma, Meilin Li, Xiangpeng Liu, Yuting Wang, Jie Zhang, Hui Shu, Zhiwei Liu, Chi Zhang, Lei Fu, Ji Hu, Yong Zhang, Zhihao Jia, Yu Feng

**Affiliations:** 1 Department of Endocrinology, The Second Affiliated Hospital of Soochow University, Suzhou, China; 2 Endocrinology Department of the Second People’s Hospital of Lianyungang City, Lianyungang, China; 3 Cambridge-Suda Genomic Resource Center, Suzhou Medical School, Soochow University, Suzhou, China; 4 Wisdom Lake Academy of Pharmacy, Xi’an Jiaotong-Liverpool University, Suzhou, China; 5 Suzhou Medical School, Soochow University, Suzhou, China

**Keywords:** Cell biology, Metabolic disorders

## Abstract

Adipose tissue, aside from adipocytes, comprises various abundant immune cells. The accumulation of low-grade chronic inflammation in adipose tissue serves as a primary cause and hallmark of insulin resistance. In this study, we investigate the physiological roles of FADD in adipose tissue inflammation, adipogenesis, and adipocyte survival. High levels of *Fadd* mRNA were observed in mitochondrial-rich organs, particularly brown adipose tissue. To explore its metabolic functions, we generated global *Fadd* knockout mice, resulting in embryonic lethality, while heterozygous knockout (*Fadd*+*/−*) mice did not show any significant changes in body weight or composition. However, *Fadd*+*/−* mice exhibited reduced respiratory exchange ratio (RER) and serum cholesterol levels, along with heightened global and adipose inflammatory responses. Furthermore, AT masses and expression levels of adipogenic and lipogenic genes were decreased in *Fadd*+*/−* mice. In cellular studies, *Fadd* inhibition disrupted adipogenic differentiation and suppressed the expression of adipogenic and lipogenic genes in cultured adipocytes. Additionally, *Fadd* overexpression caused adipocyte death in vitro with decreased RIPK1 and RIPK3 expression, while *Fadd* inhibition downregulated RIPK3 in iWAT in vivo. These findings collectively underscore the indispensable role of FADD in adipose inflammation, adipogenesis, and adipocyte survival.

## Introduction

Obesity has emerged as a global pandemic, with statistics indicating that around 13% of the world’s adult population was classified as obese in 2016 [1]. This condition is marked by the accumulation of low-grade chronic inflammation, contributing to increased rates of various metabolic syndromes, as well as elevating the risks of cardiovascular disease and type 2 diabetes (T2D) [2, 3].

Adipose tissue (AT) serves as a highly dynamic organ, playing pivotal roles in energy storage, particularly in white adipose tissue (WAT) [4]. Additionally, WAT functions as a crucial endocrine organ, regulating whole-body homeostasis [5]. For instance, WAT secretes leptin and adiponectin, which act as pro-inflammatory and anti-inflammatory adipokines, respectively [6–8]. Dysfunction in WAT leads to ectopic lipid deposition and lipotoxicity in other organs, such as pancreas, skeletal muscle, liver, and heart, contributing to metabolic diseases alongside abnormal secretion of adipokines and cytokines. Apart from pre-adipocytes and mature adipocytes, WAT comprises various immune cells, including macrophages, neutrophils, mast cells, T cells, and B cells [9]. WAT-resident immune cells play critical roles in adipocyte integrity, insulin sensitivity, and metabolic regulation [3, 10]. Among these immune cells, macrophages constitute the most abundant group within WAT, accounting for up to 40–50% of the total WAT cell count [11]. In obesity, Adipose Tissue macrophages polarize into pro-inflammatory M1-like macrophages, secreting various pro-inflammatory cytokines, including tumor necrosis factor (TNF), which impairs insulin signaling and accelerates insulin resistance progression [12–14]. Therefore, comprehending the relationship between AT function and inflammation, particularly in metabolic disorders, is crucial for developing therapeutic interventions.

FAS-associated protein with death domain (FADD) serves as a pivotal adapter in transmitting apoptotic signals by interacting with FAS and TNF receptor 1 (TNFR1) death receptors (DRs), subsequently recruiting and activating procaspase-8 to facilitate cell apoptosis [15, 16]. Thus, FADD plays a critical role in inflammation and cell death regulation. Moreover, evidence has shown FADD’s involvement in various cellular processes, including adipose metabolism. Mice carrying FADD phosphorylation mutation (FADD-D) or adipocyte-specific deletion of *Fadd* are protected from HFD-induced obesity [17]. FADD loss of function diminishes AT inflammation and enhances adipocyte fatty acid oxidation through PPAR-α activation [17]. However, it remains unclear whether FADD deficiency in adipocytes affects necroptosis, as reported in other cell types [18–20]. Hence, further investigation into the interplay between cell death and adipocyte energy metabolism mediated by FADD is warranted.

## Results

### *Fadd* is upregulated during adipocyte differentiation

**Fig. 1:**
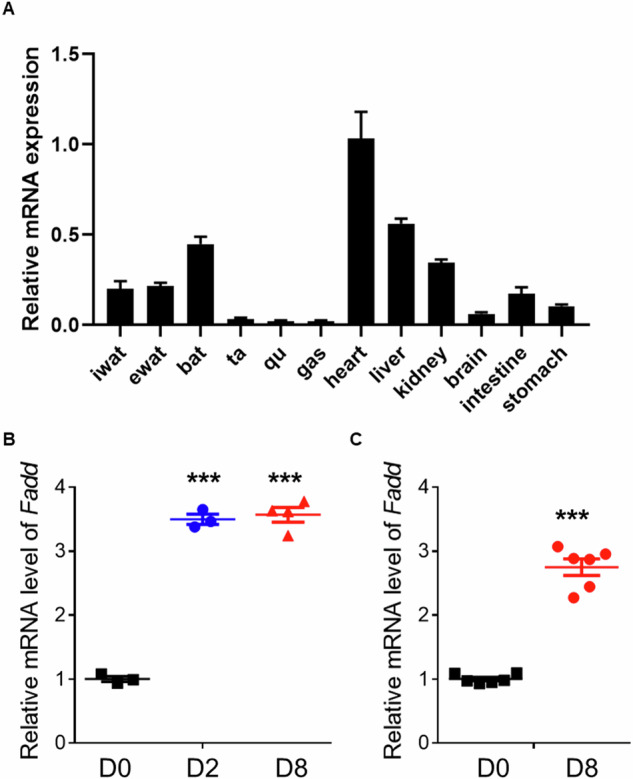
*Fadd* level is increased during adipogenesis. **A** qRT‐PCR detection of *Fadd* expression in different mouse tissues (*n* = 4). Relative levels of *Fadd* at d0 and d8 during adipogenic differentiation of preadipocytes isolated from iWAT (**B**) and BAT (**C**) (*n* = 4). Data represent mean ± s.e.m. (*t*-test: ***P < 0.001).

To investigate the potential roles of FADD in vivo, we initially assessed its mRNA levels in various tissues. *Fadd* mRNA levels were highest in the heart, followed by the liver and BAT (Fig. 1A), while they remained low in all tested skeletal muscle tissues (Fig. 1A). Subsequently, we evaluated whether the expression levels of *Fadd* were correlated with WAT and BAT adipogenesis in vitro. The expression levels of *Fadd* were upregulated in differentiated stromal vascular fraction (SVF) preadipocytes isolated from iWAT at D2 and D8 compared to undifferentiated D0 control (Fig. 1B). Similarly, mRNA levels of *Fadd* were increased by approximately three-fold in differentiated BAs (Fig. 1C). These findings collectively suggest that Fadd may play a role in WAT and BAT adipogenesis.

### Global knockout of *Fadd* causes prenatal lethality

**Fig. 2:**
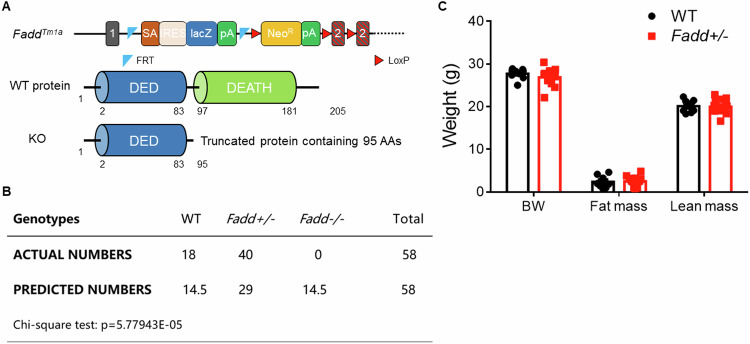
Global knockout of *Fadd* causes embryonic lethality. **A** Targeting strategy for global knockout of *Fadd*. Upper: *Fadd* gene structure showing exons (blue boxes). Middle: FADD protein domain structure with amino acid numbers labeled. Lower: excision of *Fadd* results a premature translational stop, generating a truncated protein without the key DEATH domain. **B** Chi-square test of the actual and predicted genotyping of 58 pups bred from *Fadd +/−* male with *Fadd +/−* female. **C** Body weight, fat mass, and lean mass of the mice as detected by an Eco-MRI, *N* = 6 WT mice and 11 *Fadd +/−* mice.

We then injected a mutant mouse ES cell obtained from the ES cell bank of CAM-SU, which contained a modified *Fadd* genome region to induce *Fadd* knockout. The homozygous knockout alleles resulted in a premature translational stop and the generation of a truncated peptide lacking the key DEATH domains of FADD (Fig. 2A). Mouse genotyping revealed the absence of homozygous knockout mice, and a Chi-square test calculation of littermates indicated that homozygous knockout of *Fadd* led to prenatal lethality (Fig. 2B). Therefore, we proceeded to analyze the phenotype of heterozygous knockout (*Fadd*+*/−*) mice. At 10 weeks of age, there were no discernible differences in body weight, fat mass, or lean mass between wild type (WT) and *Fadd*+*/−* mice (Fig. 2C). These results collectively suggest that loss of *Fadd* leads to prenatal lethality in mice, and heterozygous knockout of *Fadd* has minimal impact on body composition.

### Inhibition of *Fadd* promotes global energy metabolism of mice

**Fig. 3:**
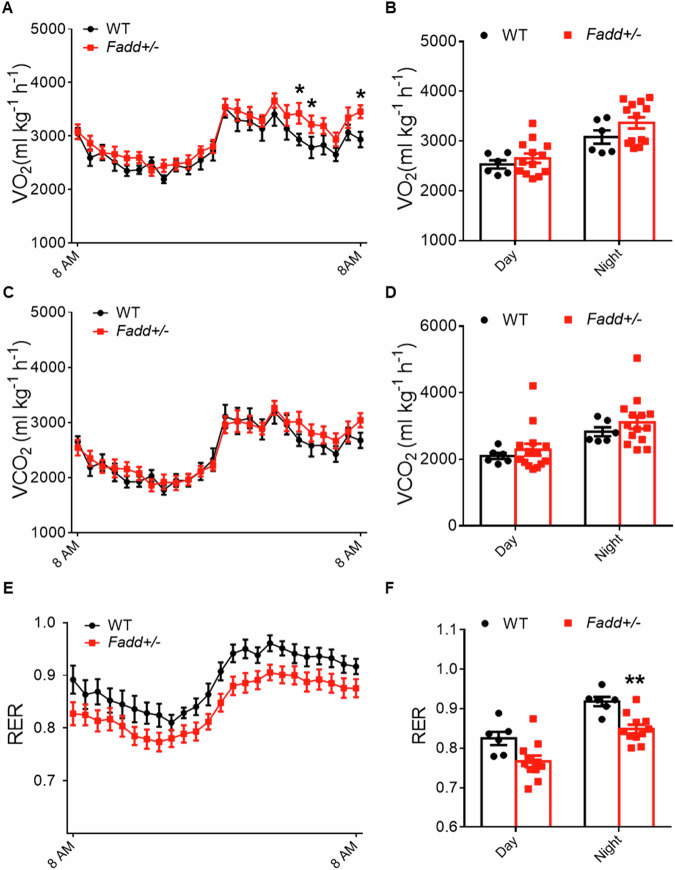
Heterozygous knockout of *Fadd* lowers respiration exchange rate. O_2_ consumption (**A**), average day and night O_2_ consumption (**B**), CO_2_ production (**C**), average day and night CO_2_ consumption (**D**), RER (**E**, **F**), and average day and night O_2_ RER (**F**) of 12-week-old male WT and *Fadd +/−* mice are measured by an indirect calorimetry, *N* = 6 WT mice and 14 *Fadd +/−* mice. Data represent mean ± s.e.m. (*t*-test: **P < 0.01).

**Fig. 4:**
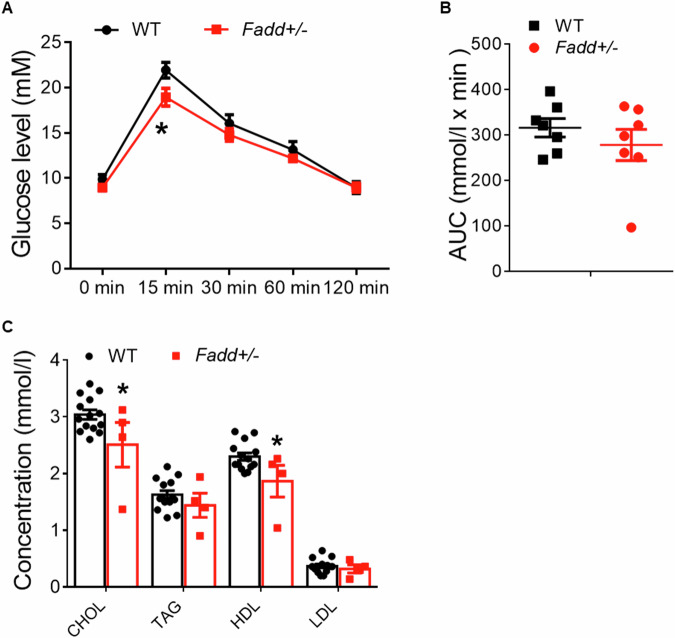
Heterozygous knockout of *Fadd* promotes glucose tolerance and lowers serum lipid. Blood glucose concentrations during glucose tolerance test (GTT) performed on mice after 12-week-old WT and *Fadd +/−* mice (**A**) and area under curve (AUC) (**B**), *N* = 7. **C** Concentrations of Cholesterol, HDL, LDL, and TAG from the serum of WT and *Fadd +/−* mice, *N* = 14 and 4. Data represent mean ± s.e.m. (*t*-test: *P < 0.05).

We then investigated how *Fadd* loss of function affected systemic metabolism in mice. Initially, we conducted treadmill test and found no differences in oxygen consumption, carbon dioxide production, or RER (respiratory exchange ratio) between *Fadd*+*/−* mice and WT mice (Fig. S1A–C). However, oxygen consumption levels were notably higher in *Fadd*+*/−* mice compared to WT mice (Fig. 3A, B), particularly during nighttime (Fig. 3A, B), while carbon dioxide production showed no significant difference (Fig. 3C, D). Calculation of the RER revealed significantly reduced levels in *Fadd*+*/−* mice, especially during nighttime (Fig. 3E, F). As RER reflects the mice’s preference for sugar or fat as fuel, we then assessed glucose and lipid metabolism through GTT and blood biochemistry. In the i.p. GTT, the initial glucose levels of *Fadd*+*/−* mice at 15 min post-injection were significantly lower than those of WT mice (Fig. 4A), although there were no differences in the calculated area under the curve (AUC) (Fig. 4B). Blood biochemistry results indicated significantly reduced concentrations of total serum cholesterol and HDL-cholesterol in *Fadd*+*/−* mice compared to controls (Fig. 4C). These findings collectively suggest that inhibition of *Fadd* promotes systemic energy metabolism, particularly lipid metabolism.

### *Fadd* inhibition reduces adipose tissue mass and promotes adipose inflammation

**Fig. 5:**
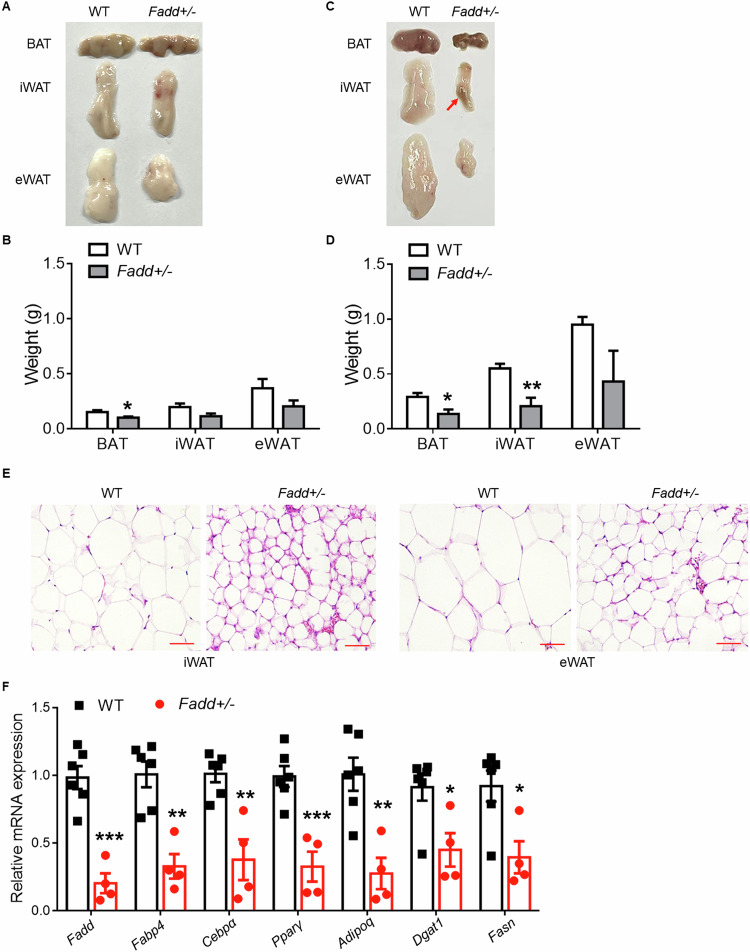
Inhibition of *Fadd* causes progressive reduction of adipose tissue mass. Represent image (**A**) and weights (**B**) of BAT and various WAT (including epididymal White Adipose Tissue, eWAT and inguinal White Adipose Tissue, iWAT) depots from 3-month-old male WT and *Fadd +/−* mice, *N* = 4. Represent image (**C**) and weights (**D**) of BAT, iWAT, and eWAT depots from 5-month-old male WT and *Fadd +/−* mice, *N* = 4. **E** Representative H&E staining image of iWAT and eWAT from 5-month-old male WT and *Fadd +/−* mice, Bar = 100 μm. **F** Relative levels of *Fadd* and genes involved in TAG synthesis and Adipogenesis iWAT of WT and *Fadd +/−* mice, *N* = 6 and 4. Data represent mean ± s.e.m. (*t*-test: *P < 0.05, **P < 0.01, ***P < 0.001).

It has been reported that *Fadd* mutation or adipocyte-specific *Fadd* deletion reduces AT mass [17]. Therefore, we aimed to investigate whether heterozygous knockout of *Fadd* also affected AT mass. At 3 months of age, the mass of brown adipose tissue (BAT) was significantly decreased in *Fadd*+*/−* mice compared to WT mice (Fig. 5A, B). Additionally, at 5 months of age, both inguinal white adipose tissue (iWAT) and BAT masses were significantly reduced in *Fadd*+*/−* mice compared to WT mice (Fig. 5C, D). H&E staining revealed that adipocyte sizes were smaller in iWAT and eWAT of WT mice compared to *Fadd*+*/−* mice (Fig. 5E). We further analyzed the expression levels of key genes involved in adipogenesis and lipogenesis using qPCR. We confirmed that mRNA levels of *Fadd* were significantly reduced in iWAT of *Fadd*+*/−* mice compared to WT mice (Fig. 5F). Moreover, the expression levels of adipogenic genes (*Fabp4*, *Cebpα*, *Pparγ*, and *Adipoq*) and lipogenic genes (*Dgat1* and *Fasn*) were all downregulated (Fig. 5E). Additionally, the masses of iWAT and epididymal white adipose tissue (eWAT) were significantly reduced in *Fadd*+*/−* mice after 7 days of cold treatment (Fig. S2).

**Fig. 6:**
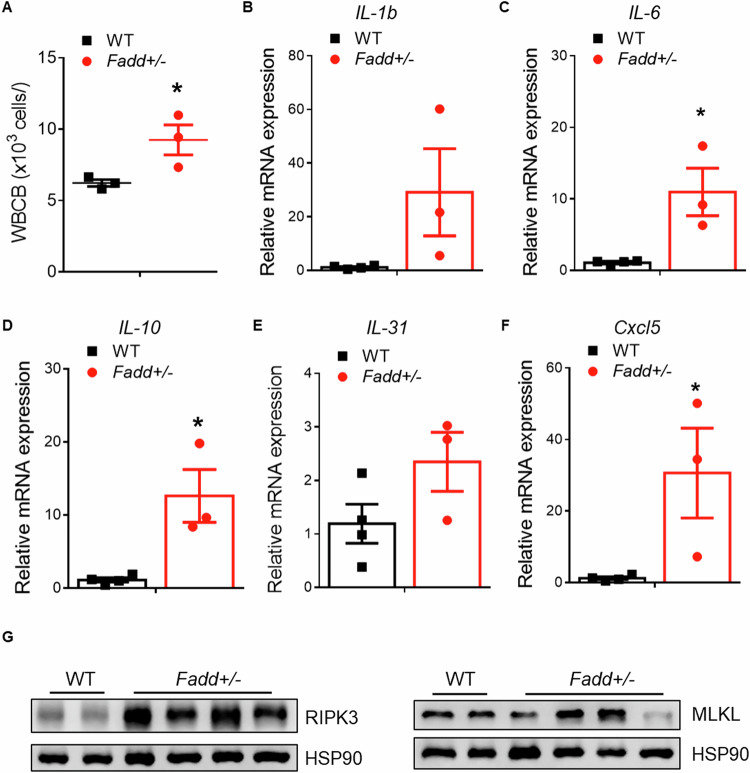
Inhibition of *Fadd* promotes global and adipose inflammatory response. **A** WBCB from the blood of WT and *Fadd +/−* mice, *N* = 3. **B**–**F** Relative levels of *IL-1b*, *IL-6*, *IL-10*, *IL-31,* and *Cxcl5* genes from iWAT of WT and *Fadd +/−* mice, *N* = 4 and 3. **G** Relative protein levels of PIPK3 and MLKL in WT and *Fadd +/−* mice, *N* = 2 WT mice and 4 *Fadd +/−* mice. Data represent mean ± s.e.m. (*t*-test: *P < 0.05).

We then investigated the underlying cause of the progressive reduction in AT mass observed in *Fadd*+*/−* mice. FADD is recognized as a key regulator of cell apoptosis and is implicated in various aspects of inflammation [15, 18]. Additionally, we noted enlarged lymph nodes in iWAT of 5-month-old *Fadd*+*/−* mice compared to WT mice (Fig. 5C). Moreover, H&E staining revealed signs of blood cell infiltration into ATs from *Fadd*+*/−* mice (Fig. 5E). Consequently, we collected blood samples from WT and *Fadd*+*/−* mice and conducted a hematology test. Fadd inhibition led to an increase in basophil white blood cells (WBCB) in mouse blood (Fig. 6A). We further examined the expression levels of inflammatory factors, including both anti- and pro-inflammatory cytokines (Fig. 6B–F). The relative mRNA levels of *Il-6* and *Il-10* were elevated in iWAT of *Fadd*+*/−* mice compared to WT mice (Fig. 6C, D). Additionally, the expression level of *Cxcl5*, a member of the C-X-C motif chemokine ligand (CXCL) family, was significantly increased in iWAT following *Fadd* inhibition (Fig. 6F). However, other interferon-inducing factors, such as *Ifit3* and *Cxcl9*, were found to be downregulated (Fig. S3). In addition, we ran WB and checked the protein levels of MLKL, and RIPK3 from iWAT of *Fadd*+*/−* and WT mice. The results showed that protein levels of RIPK3 in iWAT were significantly increased after *Fadd* inhibition (Fig. 6G). Taken together, these findings suggest that *Fadd* inhibition reduces AT mass, activates inflammatory signaling pathways, and upregulates RIPK3.

### Preadipocytes from *Fadd* +/− mice have reduced adipogenic ability

**Fig. 7:**
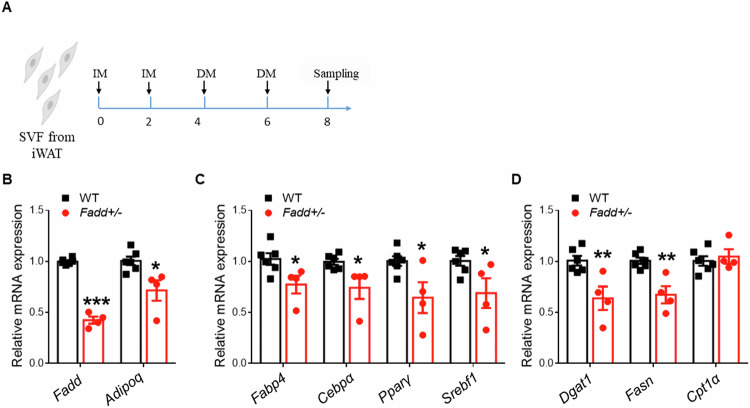
Inhibition of *Fadd* blocks adipogenesis in vitro. **A** A model depicting the process for SVF differentiation in vitro. **B**–**D** Relative levels of *Fadd* and genes involved in TAG synthesis, Adipogenesis, Lipolysis, and β-oxidation in 8-day differentiated SVF pre-adipocytes isolated from iWAT of WT and *Fadd +/−* mice, *N* = 6 and 4. Data represent mean ± s.e.m. (*t*-test: *P < 0.05, **P < 0.01, ***P < 0.001).

To further confirm whether the reduced size of iWAT in *Fadd*+*/−* mice was attributed to diminished adipogenic ability or heightened inflammation, we isolated and differentiated preadipocytes from *Fadd*+*/−* and WT mice (Fig. 7A). Following 8 days of differentiation in induction medium (IM) and differentiation medium (DM), cells were harvested for subsequent RNA extraction and qPCR analysis (Fig. 7A). As anticipated, we observed a ~ 50% reduction in Fadd mRNA levels, accompanied by downregulation of the marker gene for adipogenesis, *Adiponectin* (Fig. 7C). Additionally, relative mRNA levels of adipogenic genes (*Fabp4*, *Cebpα*, *Pparγ*, and *Srebf1*) and lipogenic genes (*Dgat1* and *Fasn*) were significantly decreased in adipocytes from *Fadd*+*/−* mice compared to WT (Fig. 7C, D). Moreover, there was no significant difference in the expression level of *Cpt1α* (Fig. 7D). These findings collectively indicate that *Fadd* inhibition impedes adipocyte differentiation in vitro.

### Overexpression of *Fadd* causes adipocyte death

**Fig. 8:**
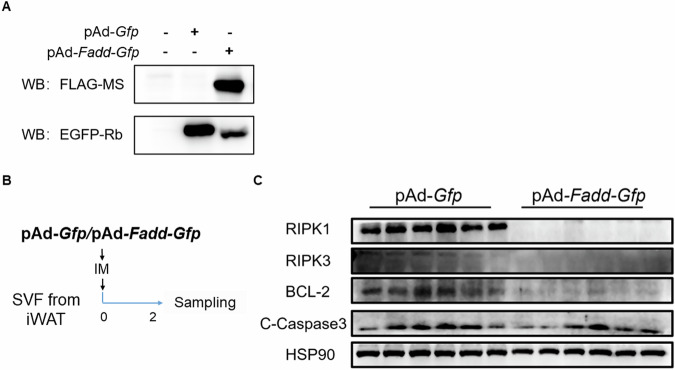
*Fadd* overexpression causes adipocyte death in vitro. **A** Western blot to verify the successful overexpression of FADD. **B** A model depicting SVF differentiation with *Ad-Fadd* in vitro. **C** Relative protein levels of RIPK1, PIPK3, BCL-2, and Cleaved-Caspase-3 in *Ad-Gfp* and *Ad-Fadd* infected adipocytes, *N* = 6.

To further investigate the role of FADD in adipogenesis, we established an adenovirus-mediated *Fadd* overexpression model. Initially, we infected 293A cells with *Ad-Fadd* and *Ad-Gfp* to validate successful overexpression. As demonstrated by GFP and FLAG blots, cells treated with *Ad-Fadd* exhibited clear FADD overexpression (Fig. 8A). Intriguingly, within 48 h of *Ad-Fadd* addition, we observed that most cells detached from the culture. Subsequently, we utilized *Ad-Fadd* virus to overexpress *Fadd* in cultured adipocytes (Fig. 8B). Protein levels of RIPK1, PIPK3, and BCL-2 were significantly reduced (Fig. 8C), while the protein level of Cleaved-caspase-3 remained unchanged (Fig. 8C). These findings suggest that *Fadd* overexpression induces adipocyte death independently of Caspase-3-mediated apoptosis.

## Discussion

Our study highlights the pivotal role of FADD in adipose inflammation, adipogenesis, and adipocyte survival. We observed high expression of *Fadd* in mitochondria-rich organs, such as BAT, with increased levels during WAT and BAT adipogenesis in vitro. Global knockout of *Fadd* resulted in embryonic lethality in mice, while heterozygous knockout led to reductions in RER and serum cholesterol levels. Moreover, heterozygous deletion of *Fadd* led to decreased AT masses in an age-dependent manner, accompanied by reduced expression levels of adipogenic and lipogenic genes in WAT. Additionally, *Fadd* inhibition promoted both adipose and global inflammatory responses. At the cellular level, heterozygous deletion of *Fadd* inhibited adipogenic differentiation by suppressing the expression of adipogenic and lipogenic genes in cultured adipocytes. Conversely, virus-mediated *Fadd* overexpression induced adipocyte death. These findings collectively underscore the indispensable role of FADD in adipose inflammation, adipogenesis, and adipocyte survival.

FADD is recognized as a key regulator of programmed cell death, with less understood functions in other biological processes, particularly metabolism [21–23]. Previous studies have shed light on the metabolic regulatory role of FADD in energy metabolism, including in AT lipid metabolism [17]. FADD is believed to be mediated through its phosphorylation at serine 191 [17, 24, 25]. However, whether this metabolic regulation mediated by FADD is attributed to altered AT inflammation remains unclear. In our current study, we demonstrated that FADD was associated with metabolic benefits and inflammation in a dose- and age-dependent manner. In 3-month-old *Fadd*+*/−* mice, we observed significantly enhanced global lipid metabolism, while 5-month-old *Fadd+/−* mice exhibited upregulated global and adipose inflammation. These findings contrast with a previous report where adipocyte-specific deletion of *Fadd* resulted in reduced AT macrophage content and expression levels of pro-inflammatory genes. Chronic AT inflammation, induced by immune cell infiltration-mediated pro-inflammatory effects, is recognized as one of the main contributors to insulin resistance [26, 27]. FADD plays a critical role in the apoptosis and inflammatory function of macrophages, and deletion of *Fadd* in macrophages has been shown to promote systemic inflammation with elevated inflammatory cytokines [28–30]. Hence, it is plausible that the AT inflammatory effect resulting from *Fadd* inhibition is a consequence of accumulated pro-inflammatory effects of AT resident immune cells, particularly macrophages. Moreover, the reduction in ADIPONECTIN secretion also exacerbates AT inflammation. Future studies employing a macrophage-specific *Fadd* KO mouse model would be ideal to further elucidate the role of FADD in AT inflammation. Additionally, the previous study utilized a *Fabp4*-driven *Fadd* deletion to investigate the function of adipocyte FADD. However, aside from potential leaking Cre expression in non-adipocytes [31–33], lineage tracing experiments also indicate that *Fabp4*-cre expression marks a population of adipogenic progenitors in the SVF from both WAT and BAT [34]. Thus, it cannot be ruled out that the reduced AT mass observed in previous reports and our current study was due to impaired adipogenesis or adipocyte metabolism. To address this, we isolated SVF preadipocytes from iWAT of control and *Fadd*+*/−* mice, cultured and differentiated them in vitro. The results suggest that heterozygous deletion of *Fadd* indeed inhibits adipocyte differentiation. Therefore, it remains unclear whether the reduced AT mass is attributable to impaired adipogenesis, inflammation, or enhanced lipid utilization. Hence, utilizing a more specific *adiponectin*-Cre to study the function of FADD in AT metabolism is essential for future investigations [35, 36].

FADD recruits and binds with initiator caspase, Caspase-8, to form the death-inducing signaling complex (DISC) during CD95-mediated apoptosis [37, 38]. In our study, we observed that protein levels of Cleaved-Caspase-3 remained unchanged in *Fadd*-overexpressing adipocytes, suggesting that FADD-mediated adipocyte death is independent of the Caspase-3 signaling pathway. BCL-2, belonging to the Bcl-2 family of anti-apoptotic proteins, regulates mitochondrial membrane permeability and facilitates apoptosis by directly releasing cytochrome c [39, 40]. We also noted decreased protein levels of BCL-2 following *Fadd* overexpression, supporting enhanced adipocyte apoptosis. Moreover, FADD serves as a suppressor of necroptosis, which is triggered by DRs and pathogen recognition receptors [19, 23, 41–43]. Necroptosis relies mainly on the interaction between receptor-interacting serine/threonine-protein kinase (RIPK)1 and 3. RIPK3 phosphorylates mixed lineage kinase domain-like protein (MLKL), initiating its translocation to the cell membrane and disrupting membrane integrity [44]. FADD forms a complex with Caspase-8 and cellular FLICE-like inhibitory protein (cFLIP), inhibiting RIPK signaling-mediated necroptosis [30]. Loss of *Fadd* in T cells leads to necroptosis during cell proliferation [41]. In our study, we observed that protein levels of both RIPK1 and RIPK3 were significantly decreased after *Fadd* overexpression, while RIPK3 was increased from iWAT of *Fadd*+*/−* mice. Moreover, knockdown of *Ripk1* inhibits the proliferation of bone marrow mesenchymal stem cells (MSCs) and leads to apoptotic cell death [45]. Since MSCs share similarities with SVF pre-adipocytes in preserving adipogenic potentials, we speculated that *Fadd* overexpression may cause a Caspase-3-independent apoptotic cell death in differentiating adipocytes. However, further investigation is required to elucidate the downstream signaling of FADD-mediated adipocyte death.

In conclusion, we have uncovered the crucial roles of FADD in AT inflammation, adipogenesis, and adipocyte survival. Therefore, targeted inhibition of FADD in adipocytes emerges as a promising strategy for combating obesity and insulin resistance. Small molecule inhibitors of FADD kinase activity, such as NSC 47147 and SP600125, present themselves as potential candidates for anti-obesity and diabetes drugs [46, 47]. However, several considerations need to be addressed, including determining the optimal dosage, duration of inhibition, and the half-life of these drugs, especially given the dose-dependent nature of FADD effects. Additionally, further efforts should focus on developing drug delivery strategies that enable controlled and targeted inhibition of FADD in AT while minimizing any impact on AT inflammation.

## Materials and methods

### Animal care

The experimental mice utilized in this study all possessed a pure C57BL/6N background and were bred and housed within the animal facility of CAM-SU (Suzhou, China). They had unrestricted access to acidified water and standard rodent chow food (radiated and autoclaved). Mouse maintenance and experimental procedures adhered to protocols approved by the CAM-SU Animal Care and Use Committee under the protocol number ZJ-2021-1 approved on December 24th, 2021. All animals were randomized picked according to the genotyping.

*Fadd* global knockout mice (Knockout First) were generated by injecting mutant mouse ES cells (containing cassettes with mouse En2 SA, LacZ, Neo, FRT, and loxP sites inserted in introns) into mouse blastocysts, followed by implantation into mouse oviducts during phantom pregnancy. Chimeric mice were then sequenced, and those with positive insertions were bred with WT C57BL/6N mice to produce *Fadd* Tm1a mice (referred to as *Fadd*−/−).

### Preadipocyte isolation and adipogenic differentiation in vitro

iWAT and BAT SVF were dissected from WT mice and digested using collagenase I, followed by density separation to isolate preadipocytes. In brief, iWAT and brown adipose tissue (BAT) were minced and digested in 1.25 mg/ml collagenase I at 37 °C for 45 min. Digestion was terminated by adding DMEM containing 20% FBS before centrifuging to remove undigested tissues. Cells were then centrifuged at room temperature at 1700 rpm for 5 min, and SVF preadipocytes were obtained in the pellet. Freshly isolated SVF cells were seeded and cultured in growth medium containing DMEM, 20% FBS, and 1% penicillin/streptomycin (P/S) at 37 °C with 5% CO_2_ until reaching 100% confluence. For adipogenic differentiation, the growth medium was replaced with (IM, 10% FBS, 2.85 mM insulin, 0.3 mM dexamethasone, 1 mM rosiglitazone, and 0.63 mM 3-isobutyl-methylxanthine in DMEM) for 4 days, followed by differentiation in differentiation medium (DM, 10% FBS, 200 nM insulin, and 10 nM T3 in DMEM).

### Body composition measurement

Total body fat and lean mass in live animals without anesthesia were measured using a Minispec LF50 body composition analyzer located in the Animal Facility of CAM-SU. Animals were gently placed in a specially sized, clear plastic holder without sedation or anesthesia. The holder was then inserted into a designated tubular space on the side of the Minispec LF50 system. To ensure accuracy, animals were kept still inside the holder during the scanning process. Each scan lasted approximately 2 min.

### Indirect calorimetry and body composition measurement

Oxygen consumption (VO_2_) and carbon dioxide production (VCO_2_) of WT and *Fadd*+*/−* mice were determined using an indirect calorimetry system (Oxymax, Columbus Instruments) located in the CAM-SU animal facility. The system maintained stable environmental conditions, including a temperature of 24 °C and humidity, with a 12 h light (8 A.M.–8 P.M.) and 12 h dark (8 P.M.–8 A.M.) cycle. Mice were individually housed in each chamber of the indirect calorimetry system with ad libitum access to food and water. Prior to the experiments, mice were acclimated to the chambers for 24 h. Energy expenditure levels were presented as averages corrected to the body weight of the mice. The average energy expenditure values during the day (8 A.M.–8 P.M.) and night (8 P.M.–8 A.M.) periods were calculated as the mean values of all points measured during the respective 12 h periods.

### Treadmill

Mice underwent a 3-day adaptation period on a treadmill set at a constant 0.7 mA electric shock and a 15% incline before the actual test, during which they were acclimated to running at a speed of 10 m/min for 10 min. On the day of the experiment, mice followed a program as: they ran at 5 m/min for 5 min, with the speed increasing by 2.5 m/min every 2 min until reaching 25 m/min, maintained for 4 min. After 25 min, both the treadmill and indirect calorimetry programs were stopped, and the mice were removed. The treadmill was then cleaned with 75% alcohol.

### Glucose tolerance test (GTT)

For GTT, mice were overnight fasting for 14 h before injected with 200 mg/ml D-glucose diluted in saline (2 g/kg body weight on mice with chow diet). The blood glucose concentrations from tail were measured by a glucometer (Accu-Check Active, Roche) 15, 30, 60, and 120 min after injection. In the test, mice were blind-caged in random orders.

### Cold treatment

For cold-exposure experiments, WT and *Fadd*+*/−* mice were individually housed and subjected to cold temperatures (4 °C) in an environmental chamber for 7 days, following established protocols [48]. Littermate controls of both WT and *Fadd*+*/−* mice were kept at room temperature in the same room.

### Blood biochemistry

Blood biochemistry analysis was conducted using a clinical chemistry analyzer (Hitachi 7100). Approximately 200 μl of plasma was collected from each mouse by centrifugation at 5000 rpm for 15 min at 4 °C. If plasma samples could not be analyzed immediately, they were stored at −80 °C until analysis. Plasma samples were utilized either undiluted or diluted to a ratio of 1:2 with deionized water if the volume was insufficient.

### Hematoxylin–eosin staining

iWAT and eWAT from WT and *Fadd*+*/−* mice were fixed in 4% PFA for 48 h at room temperature. Subsequently, the tissues underwent dehydration through a gradient ethanol process, followed by rehydration and embedding into paraffin using a Leica EG1150H embedding machine. Sections of 6 mm thickness were then cut using a Leica RM2235 Manual Rotary Microtome. For H&E staining, the sections were initially deparaffinized, rehydrated in water, and dried. Hematoxylin staining of the nuclei was performed for 15 min, followed by six washes with water. Subsequently, the sections were stained with eosin for 1 min, dehydrated, and mounted. Images were captured using an OLYMPUS IX73 microscope.

### Total RNA extraction and real-time PCR

Total RNA was extracted from cells or tissues using Trizol Reagent following the manufacturer’s instructions. The purity and concentration of the extracted RNA were assessed using a spectrophotometer (Nanodrop 3000, Thermo Fisher) at 260 and 280 nm. The absorption ratios (260/280 nm) of all samples were confirmed to be ~2–3 μg of RNA, which were then reverse transcribed using random primers and M-MLV reverse transcriptase to generate cDNA. Real-time PCR was performed on a Roche Lightcycler 480 PCR System using SYBR Green Master Mix and gene-specific primers obtained from PrimerBank. The 2^−ΔΔCT^ method was employed to analyze the relative changes in gene expression, normalized against mouse β-Actin as an internal control.

### Adenovirus generation and infection

The blank adenovirus (*pAd-Gfp*) and adenovirus carrying Fadd insertion (*pAd-Fadd-Gfp*) were obtained from Genechem (Shanghai, China). Primary WAT SVF preadipocytes were infected with either pAd-*Gfp* or *pAd-Fadd-Gfp* alongside IM.

### Protein extraction and western blot analysis

Protein was extracted from homogenized liver samples with RIPA buffer (150 mM NaCl, 1% NP-40, 0.5% sodium Deoxycholate, 0.1% SDS, 50 mM Tris-HCl, pH 8.0) that contained a protease inhibitor cocktail (Sigma) and phosphatase inhibitors NaF and Na_3_VO_4_. Protein concentrations were determined using Pierce BCA Protein Assay Reagent (Pierce Biotechnology). Equal amounts of proteins from each sample were loaded for electrophoresis (Bio-Rad). Proteins were separated by SDS–PAGE, transferred to a polyvinylidene fluoride membrane (Millipore Corporation), incubated in blocking buffer (5% fat-free milk in TBS) for 1 h at room temperature (RT), then incubated with primary antibodies (Anti-GFP, 50430-2-AP and Anti-HSP90, 13171-1-AP from Proteintech; Anti-FLAG, sab4301135 from SIGMA; Anti-RIPK1, 3493, Anti-RIPK3, 10188, Anti-BCL2, 15071 and Anti- Cleaved-Caspase-3, 9664 from Cell Signaling Technology) in blocking buffer overnight at 4 °C.

### Statistical analysis

All analyses were performed using Student’s *t*-test (two-tailed). Experimental data are expressed as mean ± SEM. Comparisons with *p* values < 0.05, <0.01, or <0.001 were considered statistically significant.

### Supplementary information

Supplymental figures and legends

Original data file

## Data Availability

All data of this manuscript is included in the main text and supplementary files.
