# Supplementary material for: FADD regulates adipose inflammation, adipogenesis, and adipocyte survival
Source: Cell Death Discov. 2024 Jul 15;10:323. doi: 10.1038/s41420-024-02089-x (PMC11250791; doi:10.1038/s41420-024-02089-x)
Supplement: Supplementary file 1 — Supplymental figures and legends [file 41420_2024_2089_MOESM1_ESM.docx]

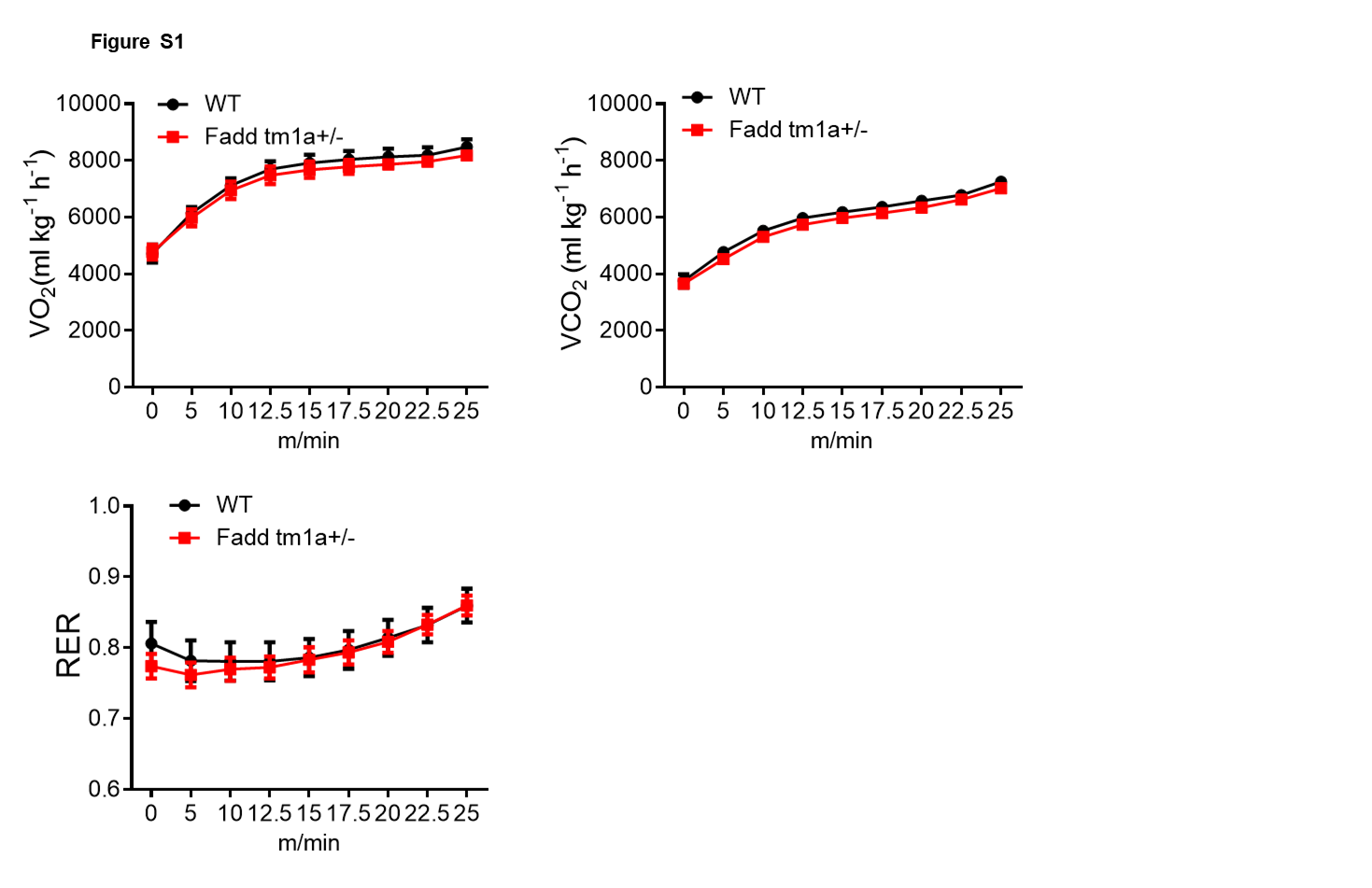


**Figure S1. Heterozygous knockout of *Fadd* overexpression did not affect exercise.**

O_2_ consumption, CO_2_ production and respiration exchange rate during exercise are measured by a treadmill incorporated with indirect calorimetry. N=8 and 9 male WT and *Fadd +/-* mice at 12-week-old.


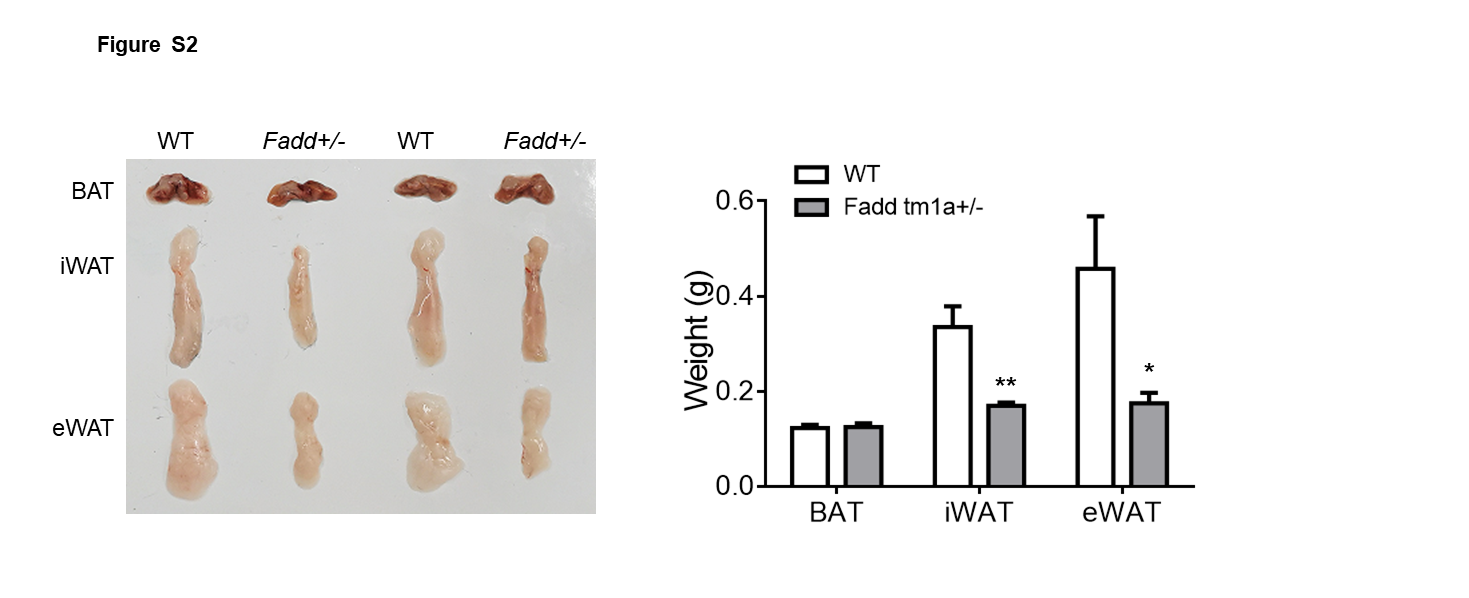


**Figure S2. Heterozygous knockout of *Fadd* lowers AT mass after cold treatment.**


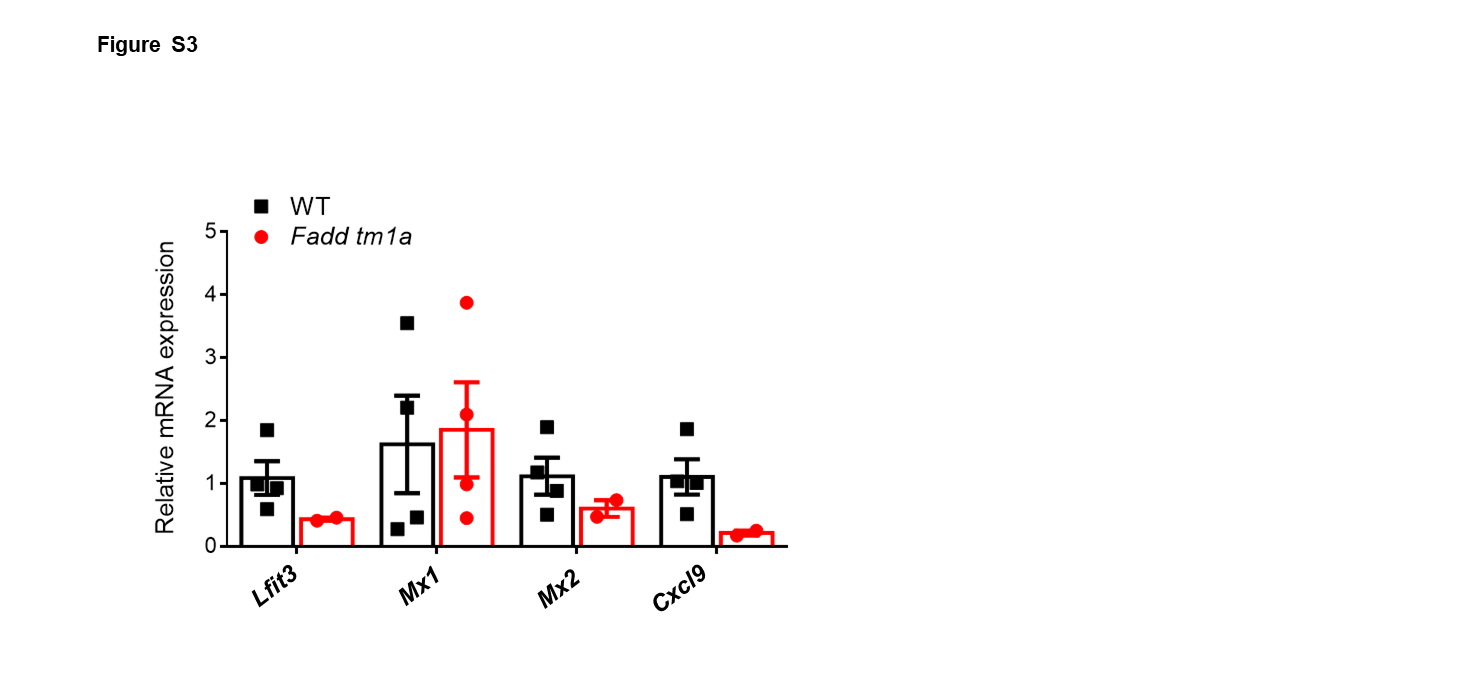
Represent image and weights of BAT and various WAT (epididymal White Adipose Tissue, eWAT and inguinal White Adipose Tissue, iWAT) depots after 7-day of cold treatment, N=4.

**Figure S3. Heterozygous knockout of *Fadd* lowers AT mass after cold treatment.**

Relative levels of *Lfit3*, *Mx1*, *Mx2* and *Cxcl9* genes from iWAT of WT and *Fadd +/-* mice, N=4 and 2.
