# Supplementary figures and images for: FADD regulates adipose inflammation, adipogenesis, and adipocyte survival
Source: Cell Death Discov. 2024 Jul 15;10:323. doi: 10.1038/s41420-024-02089-x (PMC11250791; doi:10.1038/s41420-024-02089-x)

Uncropped gel

Figure 6G

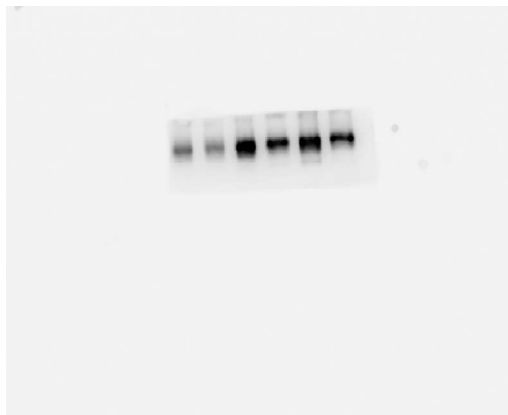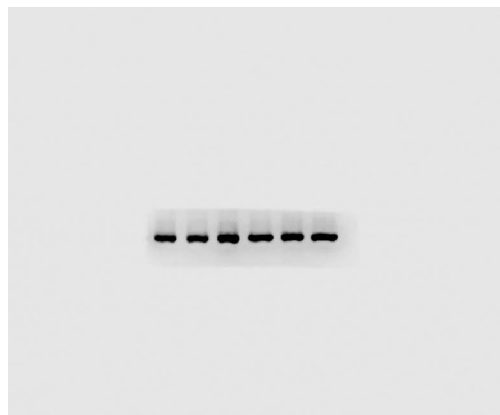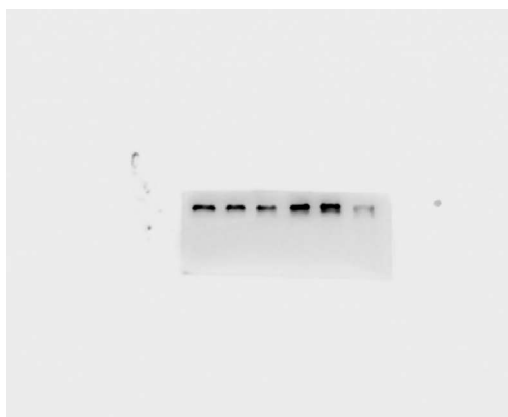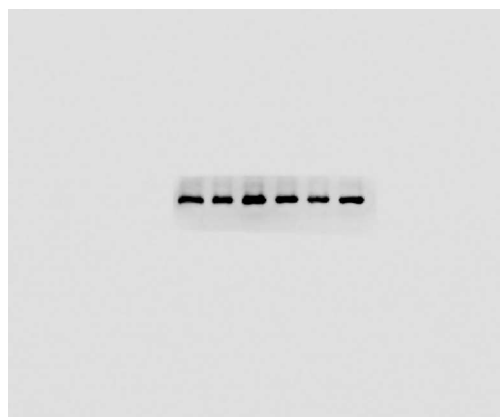

Uncropped gel

Figure 8A

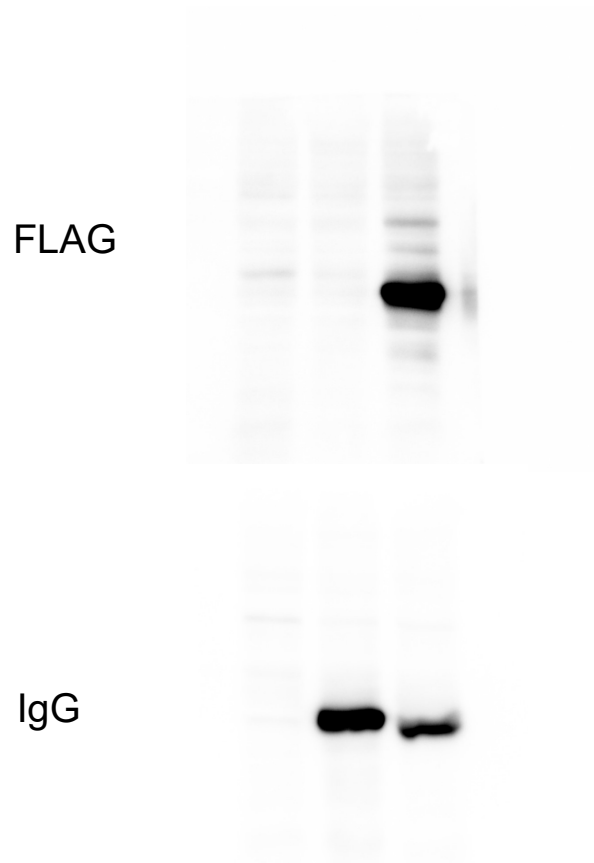

Figure 8C

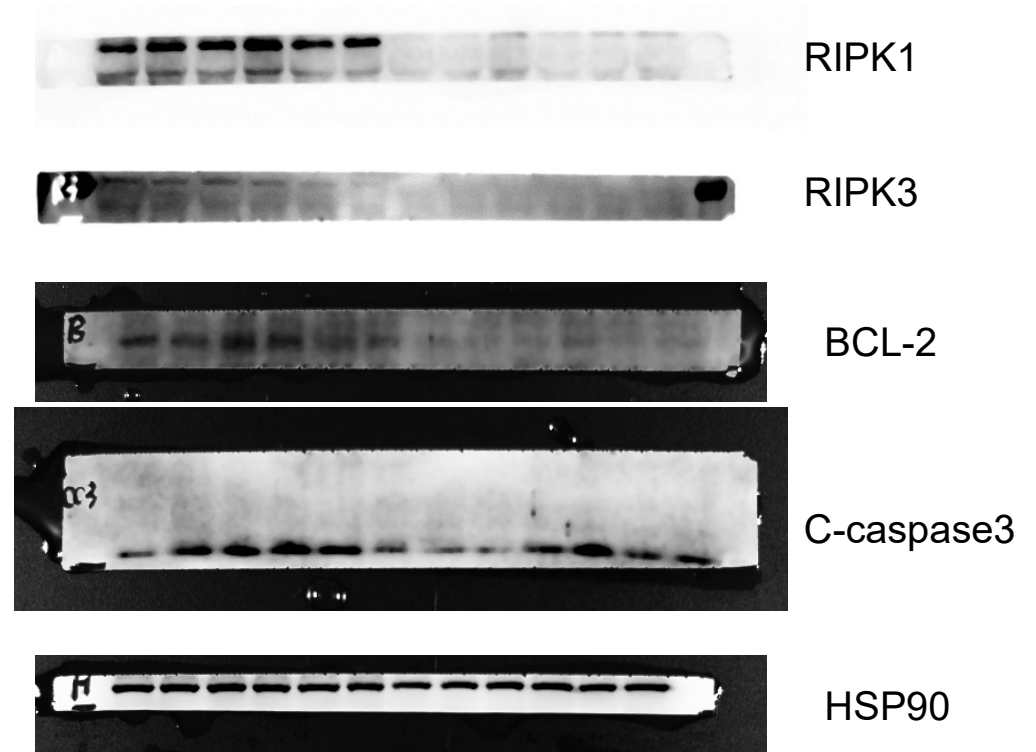

Supplement: Supplementary file 2 — Original data file [file 41420_2024_2089_MOESM2_ESM.pdf]
